# Drug reaction with eosinophilia and systemic symptoms syndrome secondary to bimekizumab

**DOI:** 10.1016/j.jdcr.2026.01.060

**Published:** 2026-02-18

**Authors:** Katharine Fray, Ilia Anna Petrou, Deirdre O'Callaghan

**Affiliations:** a University of Manchester and Salford Royal NHS Foundation Trust, Manchester, United Kingdom; b University Hospitals Sussex NHS Foundation Trust, Sussex, United Kingdom

**Keywords:** Bimekizumab, DIHS, DRESS, drug hypersensitivity, drug reaction

## Introduction

Drug reaction with eosinophilia and systemic symptoms (DRESS) is a potentially life-threatening drug-induced reaction that can present with cutaneous, hematological, and internal organ involvement. It is a T-cell–mediated delayed type IVb drug hypersensitivity reaction.^1^^,^^2^ Bimekizumab is the first monoclonal antibody to selectively inhibit interleukin (IL)-17F and IL-17A and has been approved for the management of plaque psoriasis and psoriatic arthritis in the United Kingdom and European Union.^3^

## Case report

**Fig 1:**
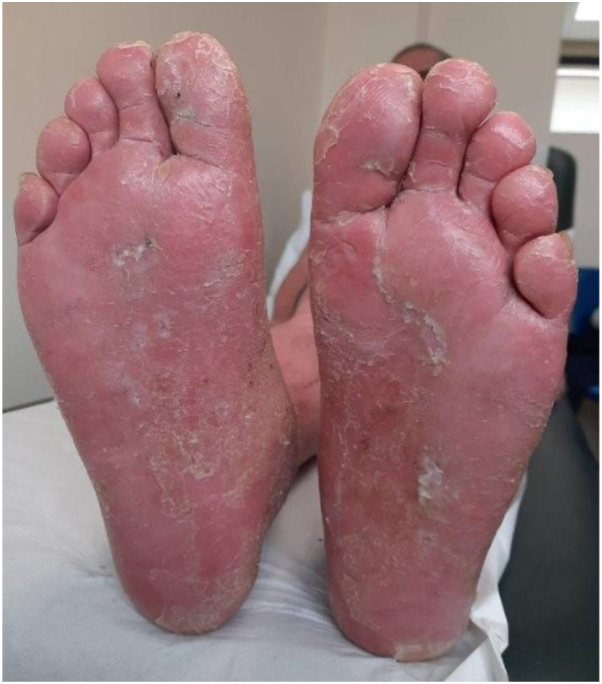
The patient had a new-onset palmoplantar disease at the initial presentation with Drug Reaction with Eosinophilia and Systemic Symptoms (DRESS). This also recurred 2 months later, alongside the erythematous rash on his legs.

**Fig 2:**
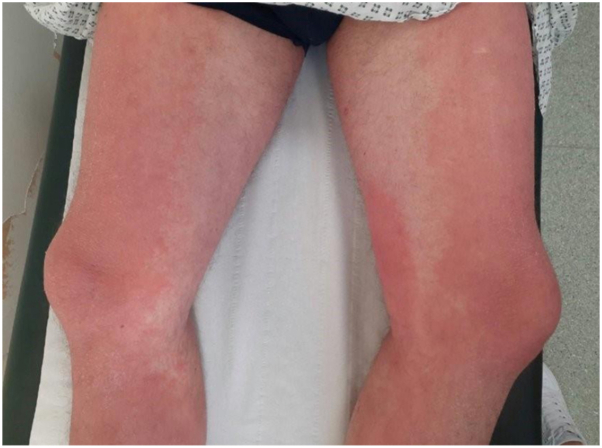
The patient presented 2 months later after the initial episode, while tapering the topical steroids, with a bright erythematous rash, which was very itchy and mildly scaly, on both anterior and posterior aspects, with a few areas of sparing.

We present a case of DRESS induced by bimekizumab, to our knowledge, previously unreported. A 45-year-old man with chronic plaque psoriasis experienced a 2-week history of worsening erythroderma with palmoplantar involvement (Fig 1), edema (including face), cervical lymphadenopathy, fever, and tachycardia. He had a widespread eczematous exfoliative rash (Fig 2), starting 10 days after his first bimekizumab injection. He was established on adalimumab for 5 years, with no other medication, but was replaced due to secondary failure. Laboratory results revealed eosinophilia, lymphocytosis with reactive forms, raised inflammatory markers, and deranged liver function. Histology showed patchy interface dermatitis, with basal vacuolation, and a moderate to severe perivascular chronic inflammatory infiltrate in the dermis with numerous eosinophils, but no psoriasiform changes. The biopsy suggested a severe drug reaction rather than psoriasis. He fulfilled the diagnostic criteria for DRESS (Registry of Severe Cutaneous Adverse Reaction [RegiSCAR] > 5, Table I). The differentials included erythrodermic psoriasis, acute generalized exanthematous pustulosis, and pityriasis rubra pilaris, but biopsy and clinical features (including absence of pustules and lack of island sparing) did not support these. The management of DRESS included discontinuation of bimekizumab, fluid resuscitation, topical steroids, and emollients. Oral steroids were avoided, given the background of psoriasis. Some improvement in his exfoliative dermatitis occurred over 4 days. Liver function and lymphocytosis normalized after 5 weeks, but the eosinophilia persisted. Tapering topical steroids worsened cutaneous symptoms. He was reviewed 7 weeks later with a persistent, eczematous, itchy rash, more demarcated and edematous on his legs, with persistent palmoplantar hyperkeratosis and milder exfoliative dermatitis on his trunk and upper limbs. A skin biopsy from the new rash on his legs showed features of chronic spongiotic dermatitis. He had ongoing mild eosinophilia and neutrophilia. This was managed as prolonged DRESS with an eczematous phenotype with oral ciclosporin—which has a role in the management of both DRESS and eczema—and topical steroids with partial response. The patient was transitioned to risankizumab (to prevent a new psoriasis flare) with synchronous tapering of ciclosporin. We chose an IL-23 biologic because the patient had previously failed adalimumab and bimekizumab. Six months later, his psoriasis was well controlled, but his eczematous rash persisted, mainly affecting the lower limbs and palmoplantar areas. Methotrexate was added for the coexisting eczematous rash on the legs, leading to gradual resolution within 4 weeks.

**Table I:** Registry of Severe Cutaneous Adverse Reaction criteria for DRESS (European Registry of Severe Cutaneous Adverse Reactions to Drugs and Collection of Biological Samples), highlighting the scoring of our case (variables with ✓):

Variable	No	Yes	Unknown
Fever (≥38.5 °C)	−1	0 ✓	−1
Enlarged lymph nodes (≥2 sites, >1 cm)	0 ✓	1	0
Atypical lymphocytes	0	1 ✓	0
Eosinophilia: 0-699 cells or <10% (no eosinophilia)		0	
Eosinophilia: 700-1499 cells or 10%-19.9%		1 ✓	
Eosinophilia: ≥1500 cells or ≥20%		2	
Acute rash: >50% of body surface area	0	1 ✓	0
Acute rash with at least 2 of: edema, infiltration, purpura, scaling	−1	1 ✓	0
Biopsy suggesting DRESS	−1	0 ✓	0
Internal organ involvement∗: 1 organ		1 ✓	
Internal organ involvement∗: 2 organs		2	
Resolution in ≥15 days	−1	0 ✓	−1
Alternative diagnoses excluded (by ≥3 biological investigations)	0	1 ✓	0

Interpretation of total score:

<2 points: no case of DRESS.

2 to 3 points: possible case of DRESS.

4 to 5 points: probable case of DRESS.

>5 points: definite case of DRESS **✓.**

*DRESS*, Drug Reaction with Eosinophilia and Systemic Symptoms.

∗ Organs involved: liver, kidney, lung, muscle/heart, pancreas, and other organ(s).

## Discussion

DRESS has an incidence of between 1 in 1000 and 10,000.^2^ This delayed T-cell–mediated type IVb drug hypersensitivity reaction involves CD4+ Th2 cells and other CD4+ and CD8+ subtypes, with an expansion of regulatory T cells.^2^ Research demonstrates a genetic component; specific HLA alleles predispose individuals to DRESS. Two diagnostic criteria are available, the International RegiSCAR group and the Japanese Consensus Group.^3^ The presentation fulfilled all but one of the RegiSCAR criteria (Table I) with only one site of lymphadenopathy rather than the criteria of 2 distinct sites.^3^ Diagnosis can be challenging due to the pattern of cutaneous involvement and the varied internal organ involvement.^4^ Mortality rates for the syndrome are up to 6.1%^2^ highlighting the importance of prompt diagnosis and management.

Common triggers of DRESS include aromatic anticonvulsants, antibiotics, dapsone, and allopurinol.^2^^,^^5^ This case highlights DRESS occurring with bimekizumab (IL-17A/IL-17F inhibitor) used for chronic plaque psoriasis. No prior published cases were found, although DRESS has been reported with guselkumab,^6^ nivolumab,^2^ and tocilizumab,^7^ raising the possibility of a rare class effect. US Food and Drug Administration review documents note one DRESS-coded event during bimekizumab development, whereas EMA’s 2024 update does not list DRESS as an adverse effect, suggesting that this reaction is exceptionally uncommon.

Generally, there is a latency period of 2 to 8 weeks, and some clinicians reject the diagnosis of DRESS in cases where symptoms occur <15 days after initial exposure. Although the RegiSCAR score does not include the delay parameter, the Japanese Consensus Group criteria include a delay of >3 weeks for diagnosis.^2^ Emerging evidence describes ”rapid-onset DRESS,‟ <15 days following initiation.^8^^,^^9^ Soria et al^8^ found a statistically significant difference between the drugs inducing reaction, where drugs such as contrast media, certain antibiotics, and BRAF inhibitors were associated with a rapid onset of symptoms, whereas anticonvulsant medications and allopurinol were associated with the classical delayed-onset presentation.^8^^,^^9^ A hypothesized mechanism is that some drugs activate the immune system faster than others. Bimekizumab is considered one of the fastest-acting biologics for psoriasis. A further challenge with our patient was the persistent eczematous rash, more prominent on the legs, the biopsy from which showed chronic spongiotic dermatitis. The histology of DRESS can show various inflammatory patterns, including eczematous and interface changes.^2^ We hypothesize that this was a manifestation of prolonged DRESS with an eczematous phenotype. The longer half-life of bimekizumab might have contributed to prolonged symptoms of DRESS. Being an IL-17 inhibitor, bimekizumab could have caused additional cytokine imbalance, allowing the emergence of T-helper 2–driven inflammation,^10^ normally suppressed by Th1/Th17 immune activity, contributing to the eczematous phenotype.

## Conclusion

This case of DRESS demonstrates a reaction to bimekizumab, initiated for treatment of chronic plaque psoriasis, previously undocumented. The onset of symptoms was <2 weeks from drug exposure. A short latent period between initiation of the culprit drug and the onset of cutaneous involvement has been described and seems to be more associated with specific drug classes, including contrast medium and antibiotics.^6^^,^^7^ Cases of other monoclonal antibodies inducing DRESS^9^ have been reported, posing the question of whether this reaction is a class effect.

## Take-Home Messages

• We described a case of prolonged DRESS due to bimekizumab, initiated for chronic plaque psoriasis.
• Rapid-onset DRESS (<15 days) is an increasingly recognized entity. It is well described with BRAF inhibitors and anakinra.
• This case highlights that biologics with longer half-lives have the potential of leading to prolonged adverse drug reactions.

## Conflict of interest

None disclosed.
